# Five seconds to safety: detecting and managing critical instability before structured handover in non-trauma patients

**DOI:** 10.1186/s13049-026-01545-0

**Published:** 2026-01-08

**Authors:** Ingmar Gröning, Karl-Christian Thies, Henning Biermann, Christoph Wasser, Sascha Ostrowski, Cordt Beißner, Bernhard Kumle, Philipp Kümpers, Michael Bernhard, Mark Michael

**Affiliations:** 1 Clinic for Emergency Medicine, Alexianer Hospital Maria Hilf, Krefeld, Germany; 2 Department of Anaesthesiology, Intensive Care, Emergency Medicine, Transfusion Medicine and Pain Therapy, Bielefeld University, Medical School and University Medical Centre OWL, EvKB, Bielefeld, Germany; 3 Department for Acute and Emergency Medicine, University Hospital RWTH Aachen, Aachen, Germany; 4 Department of Clinical Acute and Emergency Medicine, Robert Bosch Hospital Stuttgart, Stuttgart, Germany; 5 Emergency Department, Marienhospital Gelsenkirchen, Gelsenkirchen, Germany; 6 Center for Emergency Medicine, Knappschaft Kliniken Vest, Recklinghausen, Germany; 7 Clinic for Acute and Emergency Medicine, Schwarzwald-Barr Clinic, Villingen-Schwenningen, Germany; 8 Emergency Department, University Hospital Münster, Münster, Germany; 9 Emergency Department, University Hospital Düsseldorf, Heinrich Heine University Düsseldorf, Düsseldorf, Germany; 10 Resuscitation Room Working Group, German Society for Emergency Medicine (DGINA), Berlin, Germany

**Keywords:** Resuscitation room, Critically ill patients, Communication, Risk management, Quality improvement, 5-second round

## Abstract

In recent years, effective and safe patient handover concepts have been established in the resuscitation room. The utilisation of a structured scheme and the establishment of a 'handover culture', wherein all team members recognise the significance of this phase, serve as crucial milestones. In the interest of patient safety, it is recommended that a "5-second round" is conducted prior to the initiation of the handover process. The purpose of this procedure is to facilitate the evaluation of the patient's stability during the handover phase, thereby identifying the necessity for critical interventions to be performed beforehand. Originally developed for trauma patients, this tool is also relevant for critically ill non-trauma patients. The objective of this article is to provide a more detailed description of the original 5-second round, to adapt its application for use in critically ill non-trauma patients and to give some examples of concrete actions.

**Supplementary Information:**

The online version contains supplementary material available at 10.1186/s13049-026-01545-0.

## Introduction

The interface between emergency medical services (EMS) and emergency departments (ED) has become the focus of scientific research in recent decades. The structured handover of patients was described by the World Health Organization (WHO) as early as 2007 as an essential process that can significantly increase patient safety [1]. The conceptualization of handover processes was predominantly initiated within the domain of educational programs designed for the management of trauma patients in emergency rooms. Prominent examples include the Advanced Trauma Life Support (ATLS) and the European Trauma Course (ETC) curricula. For patients suffering from trauma, the MIST (Mechanism, Injury, Symptoms/Signs, Treatment) or ATMIST scheme (Age, Time, Mechanism, Injury, Symptoms/Signs, Treatment) has become established as a commonly used handover scheme, particularly in English-speaking countries [2, 3], but there is no global consensus.

The 5-second round instrument was first described in 2008 in the manual of the European Trauma Course (ETC) and referenced in subsequent publications [4, 5]. Accordingly, a "Plan A" and “Plan B”, are defined during the preparatory phase. Upon arrival, the team leader should use the 5-second round to form a concise clinical impression. to determine whether “Plan A” is safe of if the "Plan B" (escape plan) must be initiated in response to either "catastrophic hemorrhage, airway occlusion, or traumatic cardiac arrest". If the patient's condition is deemed stable (“Plan A”), a structured handover to the entire team should be performed without delay. The original ETC concept for the 5-second round proposes that a rapid initial assessment should be based on an evaluation of the three components "social interaction", "skin perfusion" and "breathing effort" as summarized in an "Assessment Triangle". The conceptual framework underlying this approach was the "Pediatric Assessment Triangle," a tool previously developed for the rapid assessment of critical conditions in children. Appearance, work of breathing and circulation to skin are three elements which allow a general impression within seconds, identifying the need for immediate intervention. If two out of the three items are found to be abnormal, a critical condition can be assumed [6].

In recent years, there has been a notable shift in scientific interest in resuscitation room care in Germany. Whereas attention was traditionally directed toward trauma patients, there is now growing emphasis on critically ill non-trauma (CINT) patients [7–10]. To provide a framework for managing CINT patients, the (PR_E-)AUD^2^IT algorithm was published as a guiding principle in 2021 [11]. This algorithm delineates a sequence of phases in the care process, including preparatory measures, primary and secondary surveys, formulation and structured evaluation of differential diagnoses and subsequent diagnostic procedures. It also outlines the interpretation of findings and subsequent therapeutic interventions, culminating in disposition decision and to-do list generation. The algorithm is interspersed with three designated team time-outs. The 5-second round is an integral component of the first team time-out and is positioned immediately prior to the handover [11].

The goal of this article is to provide an improved description of the original 5-second round and to adapt its application for the use in CINT patient care.

### 5 seconds for patient safety – is the patient stable enough for handover?

The establishment of a "handover culture" and a standardized procedure has been demonstrated to reduce information loss during handover, enhance patient safety and optimize cooperation between emergency medical services and emergency room teams [12]. The authors argue that the 5-second round is an active process by the receiving side, specifically by the team leader, and not solely an evaluation by the transferring EMS. If the receiving team does not independently initiate the 5-second round, the transferring team should explicitly request that it be performed. The EMS team should also proactively display the current vital signs on the monitor. The objective of this collaborative process is to identify fixation errors, detect previously unrecognized clinical deterioration, and ultimately improve patient safety. The 5-second round makes explicit a diagnostic process that experienced clinicians often use intuitively: the "first impression" focused on social interaction to identify life-threatening conditions. The absence of social interaction between patients and the treating team is arguably the most rapid and sensitive indicator of a life-threatening condition.

Following the initial publication of the 5-second study nearly two decades ago, a steady and consistent progression in medical technology within the field of EMS has occurred. Consequently, technical and logistical aspects can become increasingly crucial in the handover of CINT patients. For instance, unaddressed alarm messages on ventilators, such as pressure alarms, may be of particular concern. Moreover, in the event of depleted oxygen reserves, low battery levels, or depleted syringe pumps, acute actions may be required. This may necessitate adjustments to the oxygen source or the initiation of concurrent, overlapping monitoring.

### Adaption of the 5-second round to critically ill non-trauma patient care

From the authors' perspective, the 5-second round for CINT patients encompasses a few aspects that were not initially described in trauma care.

**Fig. 1:**
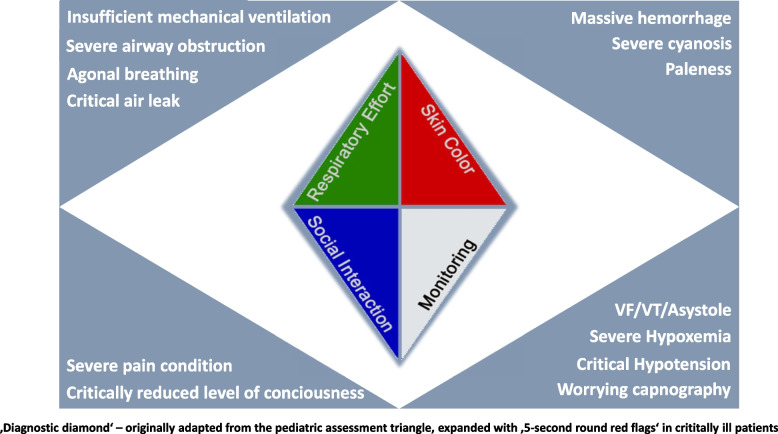
The *Diagnostic Diamond* – visualization of the main aspects of the 5-second round

Firstly, the formal incorporation of vital signs into the 5-second round is significant. The four resulting components– social interaction, respiratory effort, skin color and monitoring – can be synthesized within the framework of the 'Diagnostic Diamond' (Fig. 1) to identify "red flags" in the patient’s condition during handover.

Secondly, it is imperative that the set of indications and their respective interventions is extended to align with the typical issues that may arise in the context of CINT patient handover (Table 1). Generally, only those measures that are necessary and immediately required to stabilize the patient's condition to prevent the patient from further harm during the handover process should be addressed in this phase. It must not become a primary survey brought forward. In response to issues detected during the 5-second round, a range of possible interventions may be considered, including mainly simple procedures (e.g., nasopharyngeal tube placement), escalations of therapy (e.g., increasing oxygen flow), the administration of emergency medication prepared during the preparation phase (e.g., bolus administration of catecholamine as known as push-dose pressor). In rare cases, complex interventions e.g cardiopulmonary resuscitation as seen as an ‘escape plan’ needs a change to resuscitation algorithms. However, the decision to implement one of these measures is contingent upon the specific circumstances of each individual case, and the potential benefits for the patient must be judiciously weighed against the potential delay and disruption it may entail. In addition to the three measures previously outlined for trauma patients, the authors have consented critical interventions which, in their opinion, can be derived from a 5-second round for the treatment of CINT patients in the resuscitation room, all interventions depending on the concrete situation (Table 1). However, notably initiating a more complex intervention will likely prevent a structured handover from taking place. In such cases, the handover should be conducted to a designated member of the resuscitation room team while the others perform the intervention. Alternatively, if the EMS team (or its equipment) is involved in the complex intervention, the handover should be conducted following the successful completion of the intervention. In the absence of comprehensive data, the authors estimate that approximately 10% of cases will require any intervention prior to the initiation of the handover process.

**Table 1:** Possible interventions in the context of the 5-second round

**Common issues and potential interventions in the context of the 5-second round in critically ill non-trauma patients**		
	**Suspicion**	**Interventions**
**In case of cardiac arrest: CPR** **(change to ERC/AHA ALS-algorithm)**		
**Airway**	Obstruction from secretions	▪ Oro-, nasopharyngeal or endotracheal suctioning
	Obstruction due to tongue displacement	▪ Head tilt/chin lift/jaw thrust (Esmarch/chin-lift maneuver) or ▪ Placement of nasopharyngeal airway (Wendl tube)
	SGA/ETT dislocation	▪ Remove device, bag-valve-mask ventilation
	Critical cuff leak (SGA/ETT)	▪ Cuff reinflation *or* ▪ Remove device, bag-valve-mask ventilation
**Breathing**	Hypoxemia (low S_p_O_2_)	▪ Increase Oxygen administration (FiO_2_)
	Insufficient ventilation due to light sedation	▪ Deepening of sedation
**Circulation**	Critical hypotension (monitor)	▪ Push-dose norepinephrine ▪ Fluid aministration
	Dislocated ACCD (insufficient cardiocompression)	▪ Reposition device ▪ Consider manual compression
	Massive hemorrhage	▪ Stop the bleeding
**Disability**	Prolonged seizure/status epilepticus	▪ Consider Benzodiazepine administration if no sufficient treatment yet
**Exposure**	Severe pain condition (making transfer or handover impossible)	▪ Consider acute pain management

*Abbreviations: MV* mechanical ventilation, *SGA* supraglottic airway, *ETT* endotracheal tube, *FiO*_*2*_ fraction of inspired oxygen, *CPR* cardiopulmonary resuscitation, *ACCD* automated chest compression device

Thirdly, it is not only the initiation that should be communicated by the team leader to the entire team, but also the closure of the 5-second round. It is important to create an atmosphere of transparency and mutual respect, characterized by open dialogue and consideration for one another [13]. A typical example could be as follows:

**Table:** 

Team leader:	*"Welcome at the resus room, I am the team leader. I see the patient is awake, skin color looks ok, but breathing is laborious and oxygen saturation only 86% on 2 liters of oxygen per minute"*.
Team leader to EMS:	*"Please increase oxygen flow to 10 liters.”*
EMS:	*“Ok, oxygen is increased to 10 liters per minute.”*
Team leader to EMS:	*“No further manipulation of the patient, please start the handover!"*

Especially in cases where multiple or more complex interventions are required, the team leader has to communicate these to individual team members and/or the EMS in closed-loop communication. Example:

**Table:** 

Team leader:	*"Everyone please listen, the patient is in shock with a systolic pressure of only 75 mmHg and unresponsive. (To Nurse:) I need you to administer 10µg- of norepinephrine i.v. to maintain a systolic pressure of 90–100 mmHg."*
Nurse:	*"Understood, 10µg norepinephrine, systolic target 90–100 mmHg."*
Team leader to EMS:	*"Please take a non-invasive blood pressure measurement at short intervals and increase oxygenation with maximum oxygen flow!”*
EMS:	*"Ok. We will check the blood pressure every minute and increase oxygenation.”*
Team Leader:	*"EMS, please do not manipulate the patient any further and start the handover."*

### Implementation into clinical routine

When integrating the 5-second round into everyday clinical practice, it is important to ensure that EMS personnel do not perceive the procedure as dismissive or paternalistic. This objective can be achieved ad hoc only using appreciative and transparent communication. In the event of a coordinated interprofessional implementation, it is imperative to disseminate the underlying conceptual model through resources such as this article during the run-in phase. Furthermore, this change management process can be supported by a variety of methods, including but not limited to micro-teaching, or brief in situ refreshers during the preparatory phase. The most effective strategy is likely to be through interprofessional simulation training. This allows the 5-second round to be practiced and rehearsed using various scenarios.

In this regard, the Advanced Critical Illness Life Support (ACiLS) training concept for non-traumatic emergency room care was introduced in Germany in 2022 [14]. The course's primary focus is the (PR_E-)AUD^2^IT algorithm (see above), with the 5-second round constituting a vital element incorporated into all 16 simulation scenarios. It is also featured in the white paper on the care of CINT patients in the ED by the German Society for Emergency Medicine (DGINA), which compiles recommendations for resuscitation room care [15].

### Outlook and future direction

To date, there has been no systematic evaluation of the 5-second round. An initial step would be to analyse how often and in what form the 5-second round is performed, as well as to survey teams on their acceptance and perceived benefit for patient safety. Given the complexity of resuscitation room care, it may be difficult to attribute improvements in clinical outcomes to the 5-second round alone. Nevertheless, structured communication is widely acknowledged as a critical determinant of safety in emergency critical care [16]. The 5-second round may therefore provide a valuable tool to reduce preventable incidents during handover. Consensus methods such as a Delphi process could help refine the catalogue of critical interventions.

## Conclusion

The 5-second round translates an intuitive “first impression” into a structured and teachable practice. Extending its use to critically ill non-trauma patients offer an opportunity to strengthen patient safety during one of the most vulnerable phases of emergency care. While further evaluation is required, the concept is simple, pragmatic, and adaptable. Embedding it into education, training, and clinical standards may help it evolve from a didactic element into a routine part of resuscitation room practice.

## Supplementary Information

Supplementary Material 1.

Supplementary Material 2.

Supplementary Material 3.

## Data Availability

No datasets were generated or analysed during the current study.

## References

[1] WHO. Patient safety solutions. 2007. https://cdn.who.int/media/docs/default-source/patient-safety/patient-safety-solutions/ps-solution3-communication-during-patient-handovers.pdf?sfvrsn=7a54c664_Zugegriffen. 20.07.2025.

[2] Evans SM, Murray A, Patrick I, Fitzgerald M, Smith S, Cameron P. Clinical handover in the trauma setting: a qualitative study of paramedics and trauma team members. Qual Saf Health Care. 2010;19(6):e57. 10.1136/qshc.2009.039073. 20702445 10.1136/qshc.2009.039073

[3] Mercer S, Arul GS, Pugh HE. Performance improvement through best practice team management: human factors in complex trauma. J R Army Med Corps. 2014;160(2):105–8. 10.1136/jramc-2013-000205. 24389744 10.1136/jramc-2013-000205

[4] Thies K, Gwinnutt C, Driscoll P, Carneiro A, Gomes E, Araujo R, et al. The European trauma course–from concept to course. Resuscitation. 2007;74(1):135–41. 10.1016/j.resuscitation.2007.02.011. 17467871 10.1016/j.resuscitation.2007.02.011

[5] Thies KC, Bergmans E, Billington A, Fraga GP, Trummer F, Nasr AO, et al. The European Trauma Course: transforming systems through training. Resusc Plus. 2024;18:100599. 10.1016/j.resplu.2024.100599. 38515443 10.1016/j.resplu.2024.100599 PMC10955415

[6] Dieckmann RA, Brownstein D, Gausche-Hill M. The pediatric assessment triangle: a novel approach for the rapid evaluation of children. Pediatr Emerg Care. 2010;26(4):312–5. 10.1097/PEC.0b013e3181d6db37. 20386420 10.1097/PEC.0b013e3181d6db37

[7] Bernhard M, Doll S, Hartwig T, Ramshorn-Zimmer A, Yahiaoui-Doktor M, Weidhase L, et al. Resuscitation room management of critically ill nontraumatic patients in a German emergency department (Observe-study). Eur J Emerg Med. 2018;25(4):e9–17. 10.1097/MEJ.0000000000000543. 29406398 10.1097/MEJ.0000000000000543

[8] Bernhard M, Kumle B, Wasser C, Bergrath S, Pin M, Kümpers P, et al. Epidemiologie, Hintergründe, Zahlen Und Fakten Zum Nichttraumatologischen Schockraummanagement Kritisch Kranker Patienten. Notfall Rettungsmed. 2023;26(7):473–81. 10.1007/s10049-023-01195-0.

[9] Michael M, Kumle B, Kumpers P, Bernhard M. Management of Critically Ill Non-Traumatic Patients in the Emergency Department. Anasthesiol Intensivmed Notfallmed Schmerzther. 2022;57(7-08):466-77. Epub 20220727. 10.1055/a-1545-2422. 10.1055/a-1545-2422 35896385

[10] Michael M, Kumle B, Pin M, Kümpers P, Gröning I, Bernhard M. Nichttraumatologisches Schockraummanagement. Medizinische Klinik - Intensivmedizin und Notfallmedizin. 2021;116(5):405–14. 10.1007/s00063-021-00789-1. 33599782 10.1007/s00063-021-00789-1 PMC7891119

[11] Gröning I, Hoffmann F, Biermann H, Pin M, Michael M, Wasser C, et al. Das (Pr_E-)Aud2it-Schema Als Rückgrat Für Eine Strukturierte Notfallversorgung Und Dokumentation Nichttraumatologischer Kritisch Kranker Schockraumpatienten. Notfall Rettungsmed. 2021;25(7):491–8. 10.1007/s10049-021-00878-w.

[12] Gräff I, Pin M, Ehlers P, Seidel M, Hossfeld B, Dietz-Wittstock M, et al. Empfehlungen Zum Strukturierten Übergabeprozess in Der Zentralen Notaufnahme. Notfall + Rettungsmedizin. 2022;25(1):10-8. 10.1007/s10049-020-00810-8.

[13] Crowe L, Riley CM. Bad behavior in healthcare: an insidious threat to patients, staff, and organizations. Curr Opin Cardiol. 2024;39(4):331–7. 38547019 10.1097/HCO.0000000000001139

[14] Michael M, Biermann H, Groning I, Pin M, Kumpers P, Kumle B, et al. Development of the Interdisciplinary and Interprofessional Course Concept “Advanced Critical Illness Life Support.” Front Med. 2022;9:939187. 10.3389/fmed.2022.939187. 10.3389/fmed.2022.939187 PMC9331170 35911405

[15] Bernhard M, Kumle B, Dodt C, Gräff I, Michael M, Michels G, et al. Versorgung Kritisch Kranker, Nicht-Traumatologischer Patienten Im Schockraum. Notfall Rettungsmed. 2022;25(1):1–14. 10.1007/s10049-022-00997-y. 10.1007/s10049-022-00997-y PMC9006203 35431645

[16] Amaniyan S, Faldaas BO, Logan PA, et al. Learning from patient safety incidents in the emergency department: a systematic review. J Emerg Med. 2020;58(2):234–44. 10.1016/j.jemermed.2019.11.015. 31843322 10.1016/j.jemermed.2019.11.015

