# Supplementary figures and images for: Five seconds to safety: detecting and managing critical instability before structured handover in non-trauma patients
Source: Scand J Trauma Resusc Emerg Med. 2026 Jan 8;34:13. doi: 10.1186/s13049-026-01545-0 (PMC12849088; doi:10.1186/s13049-026-01545-0)

# Scenario 1

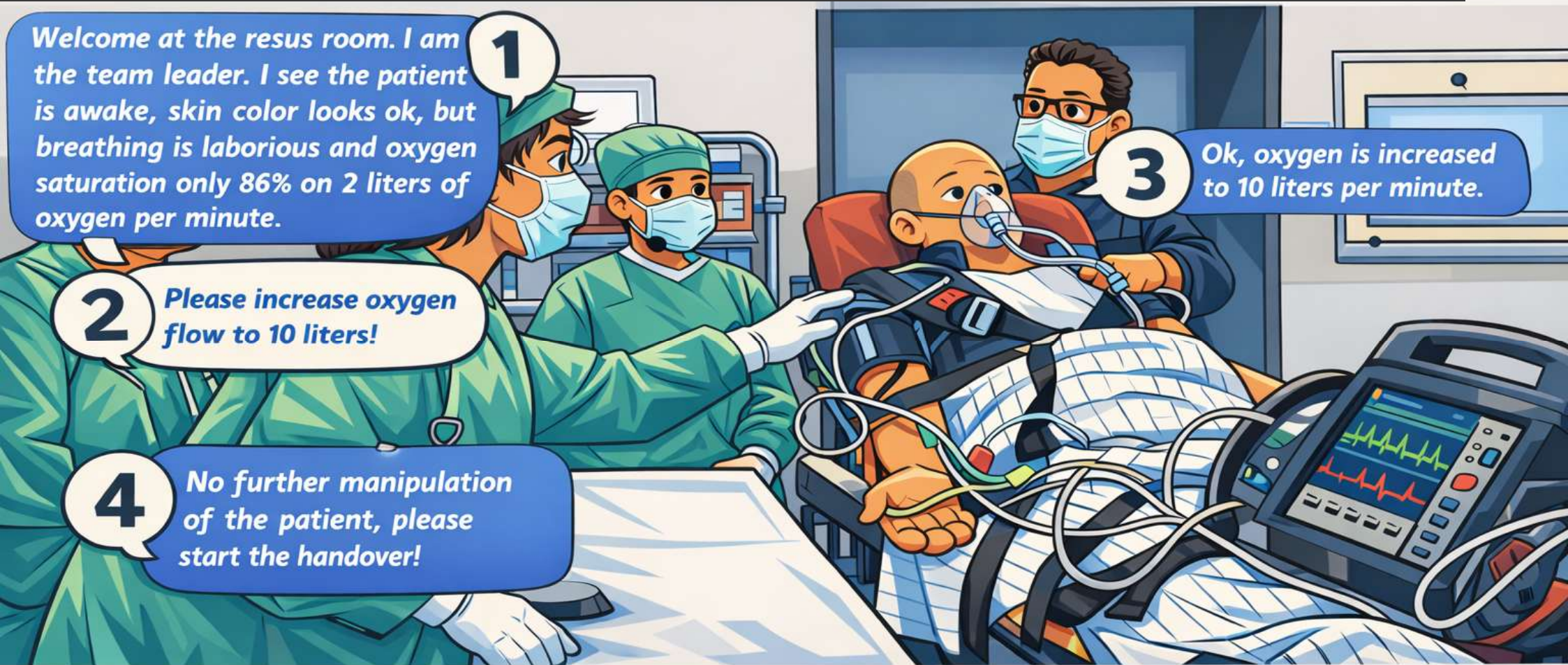

## Handover in the resuscitation room

Supplement: Supplementary file 1 — Supplementary Material 1. [file 13049_2026_1545_MOESM1_ESM.pdf]

## Scenario 2

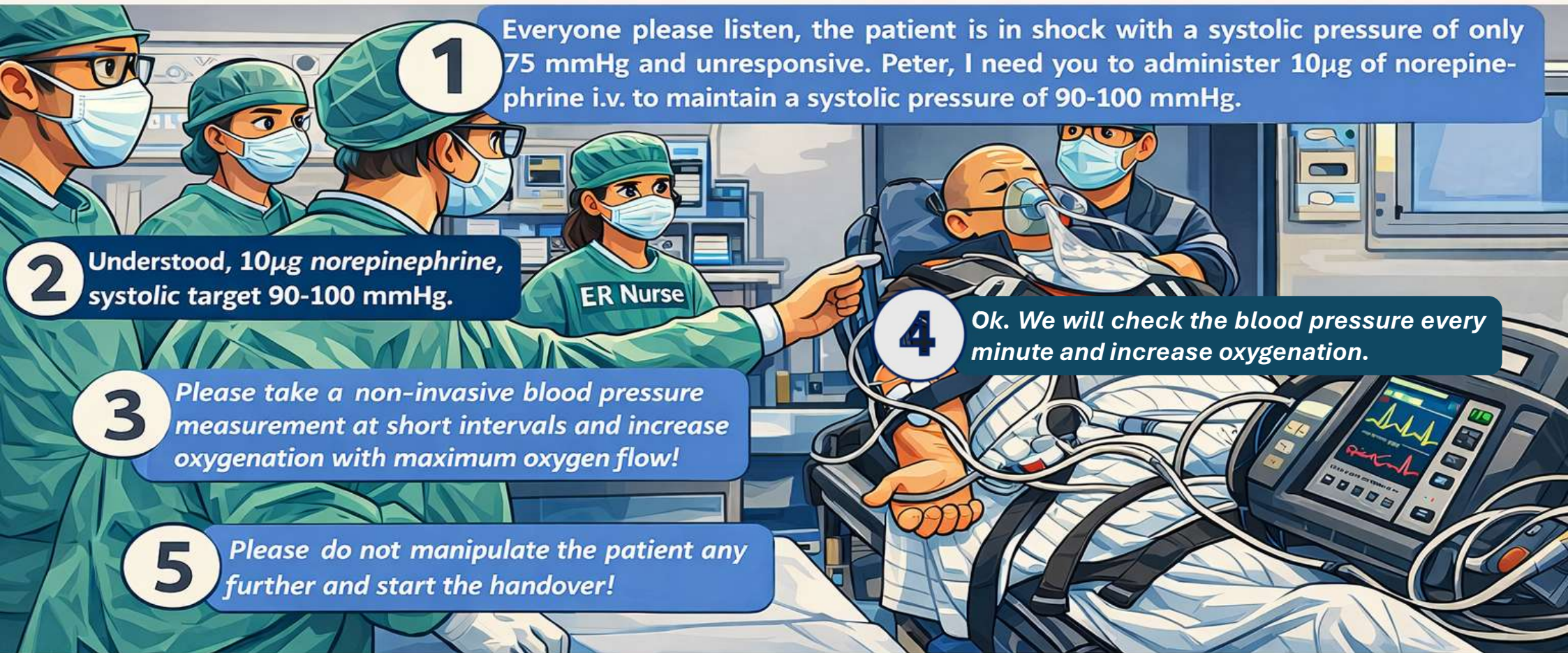

# Handover in the resuscitation room

Supplement: Supplementary file 2 — Supplementary Material 2. [file 13049_2026_1545_MOESM2_ESM.pdf]

# Scenario 3

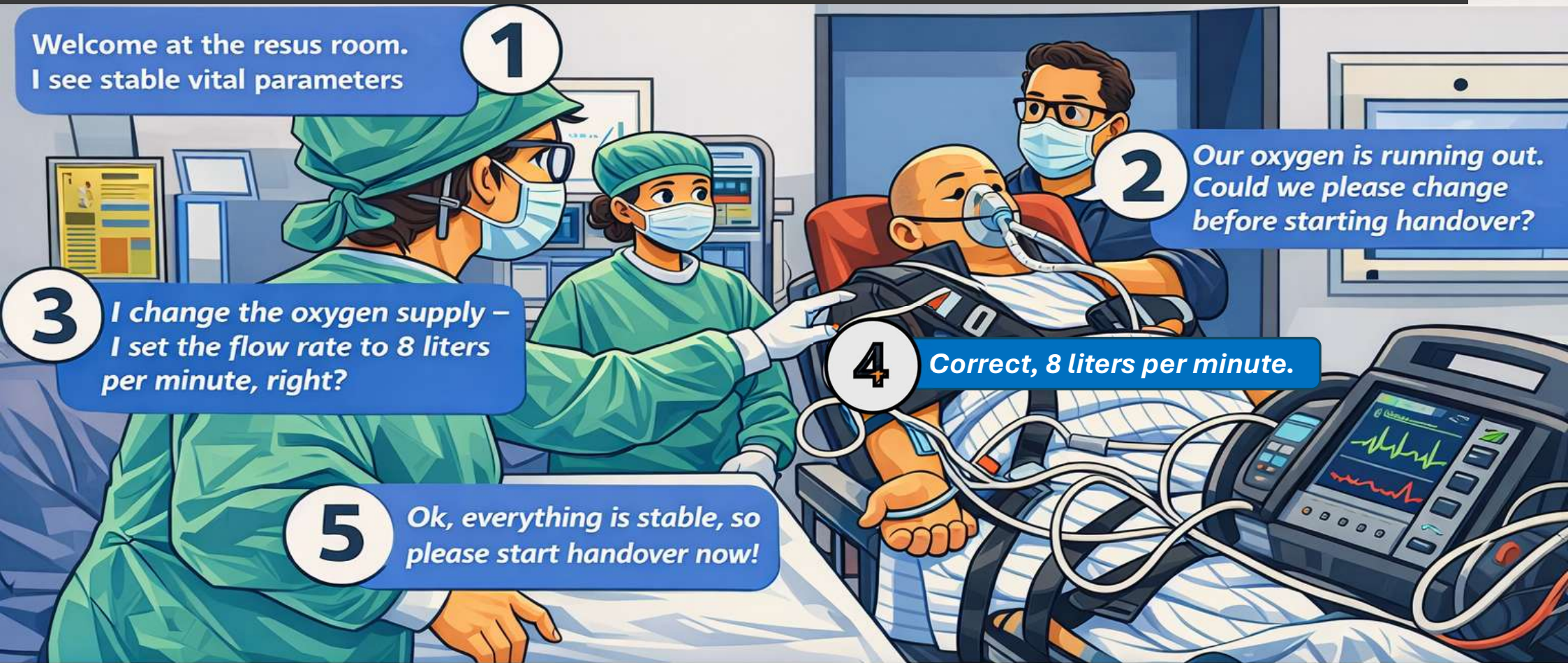

## Handover in the resuscitation room

Supplement: Supplementary file 3 — Supplementary Material 3. [file 13049_2026_1545_MOESM3_ESM.pdf]
